# Retraction: Acute Toxicity and Gastroprotection Studies of a New Schiff Base Derived Copper (II) Complex against Ethanol-Induced Acute Gastric Lesions in Rats

**DOI:** 10.1371/journal.pone.0294016

**Published:** 2023-11-10

**Authors:** The PLOS ONE Editors

Following the publication of this article [1], concerns were raised regarding reuse of results presented in Figures 2, 7, 9, and 10. Specifically,

- Figure 2B presented in this article [1] appears similar to the Control panel in Figure 2 of [2]*, and Figure 1d of [3].
- Figure 7B presented in this article [1] appears similar to Figure 5 of [4]*.
- Figure 7G of this article [1] appears similar to the following results, despite being used to represent different experimental conditions:
  - Fig. 2a of [5], when rotated
  - Figure 2a of [6, corrected in 7]
  - Figure 3 panel G1 of [8, retracted in 9], when rotated
- Figure 8F presented in this article [1] appears to partially overlap with Fig. 4D of [10, retracted in 11] when rotated.
- Figure 9F presented in this article [1] appears similar to Figure 5 panel G7 of [8, retracted in 9], despite being used to represent different experimental conditions
- Figure 10A presented in this article [1] appears similar to Figure 10G presented in this article [1] and Fig. 5A of [12, retracted in 13], despite being used to represent different experimental conditions
- Figure 10D presented in this article [1] appears similar to Fig. 4D of [14], despite being used to represent different experimental conditions

Some of the similar (or reused) images were used to represent controls in these articles, but different methodological details were reported for the indicated experiments in the articles’ Materials and Methods sections. Given the nature and extent of the issues, the *PLOS ONE* Editors are concerned about the reliability of data management and/or reporting for this study [1].

Following editorial communication regarding these concerns, one of the co-authors requested retraction of the article. The original data underlying this study were not provided for editorial review.

In light of the above concerns, the *PLOS ONE* Editors retract this article.

Some figure panels discussed above appear to report previously published material that are offered under a CC BY license, but the original article(s) was/were not attributed in [1]. For these images, the * by the citation, above, marks the oldest publication of the image of which PLOS is aware.

SG, PH, NM, and MAA agreed with the retraction. MH responded but expressed neither agreement nor disagreement with the editorial decision. NSG, AHAH, and HMA either did not respond directly or could not be reached. SG and NM apologize for the issues with the published article. MAA stands by the article’s findings.
